# Electro-echocardiographic Indices to Predict Cardiac Resynchronization Therapy Non-response on Non-ischemic Cardiomyopathy

**DOI:** 10.1038/srep44009

**Published:** 2017-03-10

**Authors:** Ziqing Yu, Xueying Chen, Fei Han, Shengmei Qin, Minghui Li, Yuan Wu, Yangang Su, Junbo Ge

**Affiliations:** 1 Department of Cardiology, Shanghai Institute of Cardiovascular Diseases, Zhongshan Hospital, Fudan University, Shanghai 200032, PR China; 2 Shanghai Medical College, Fudan University, Shanghai 200032, PR China

## Abstract

Cardiac resynchronization therapy (CRT) threw lights on heart failure treatment, however, parts of patients showed nonresponse to CRT. Unfortunately, it lacks effective parameters to predict CRT non-response. In present study, we try to seek effective electro-echocardiographic predictors on CRT non-response. This is a retrospective study to review a total of 227 patients of dyssynchronous heart failure underwent CRT implantation. Logistic analysis was performed between CRT responders and CRT non-responders. The primary outcome was the occurrence of improved left ventricular ejection fraction 1 year after CRT implantation. We concluded that LVEDV > 255 mL (*OR* = *2.236; 95*% *CI, 1.016*–*4.923*) rather than LVESV > 160 mL (*OR* = *1.18; 95*% *CI, 0.544*–*2.56*) and TpTe/QTc > 0.203 (*OR* = *5.206; 95*% *CI, 1.89*–*14.34*) significantly predicted CRT non-response. Oppositely, S wave > 5.7 cm/s (*OR* = *0.242; 95*% *CI, 0.089*–*0.657*), E/A > 1 (*OR* = *0.211; 95*% *CI, 0.079*–*0.566*), E’/A’ > 1 (*OR* = *0.054; 95*% *CI, 0.017*–*0.172*), CLBBB (*OR* = *0.141; 95*% *CI, 0.048*–*0.409*), and QRS duration >160 ms (*OR* = *0.52; 95*% *CI, 0.305*–*0.922*) surprisingly predicted low-probability of CRT non-response.

Heart failure (HF), as a common endpoint of different heart disease, brings great burden to our society^1^. To date, there is a lack of effective treatment to alleviate this disease burden. However, recent 20 years have witnessed a great progress in the area of dyssynchronous heart failure (DHF) treated with cardiac resynchronization therapy (CRT)^2^. A phenomenon that not all failing hearts assume synchronized movements makes CRT a promising way for patients with DHF. And CRT has been proved to be effective in treating DHF^3^. But, some patients whose clinical characteristics met the guidelines of CRT implantation didn’t respond to CRT^4^. Moreover, a bit of patients suffered from ventricular arrhythmia, abnormal hemodynamics, even aggravation of cardiac function after CRT implantation. Many factors are associated with CRT non-response, including aetiology of heart disease, sex, age, and cardiac function at baseline, etc^5^. Unfortunately, it still lacks of credible and convenient parameters. Electrocardiography parameters, especially QRS duration is very important to patient selection for CRT implantation^6^. Besides, other parameters reflecting the abnormal ventricular repolarization potentially predict CRT non-response^7^, e.g. QT interval, TpTe, and J wave, etc. Moreover, echocardiography dynamically presenting cardiac mechanical function makes itself conductive to predict CRT non-response^8^. To date, however, it still lacked relatively large sample size study to integratively summarize and analyze these factors mentioned above, especially for Chinese. In this study, electrocardiography, echocardiography, and blood bio-marker indices were combined to comprehensively find out CRT non-response predictors in a relatively large Chinese cohort.

## Methods

### Study population

This was a retrospective clinical study to review the patients of DHF underwent CRT implantation (n = 362) in our hospital from February 2013 to February 2016. Total 227 patients were finally included in this study, with informed consent being obtained from all subjects, while other 135 patients were excluded based on following exclusion criteria. The inclusion criteria were: (1) patients ranging from 18 to 85 years old; (2) diagnosed as decompensated heart failure with non-ischemic aetiology; (3) with LVEF less than 35%, with complete left bundle branch block (CLBBB), or with QRS more than 130 ms^9^,^10^; (4) having guideline based optimal medical treatment for at least 3 months (medical therapy was conducted by related guideline^11^, and each kind of medication was prescribed with its maximal dose that the patient could tolerate); (5) with New York Heart Association (NYHA) class II or higher levels (NYHA IV is acceptable after careful assessment of patient’s condition). The exclusion criteria were as follows: (1) incomplete clinical history record (n = 20); (2) upgraded to dual-chamber pacing with a prior right ventricular pacemaker (n = 7); (3) without follow-up clinical data especially echocardiogram because of any reason (n = 25); (4) confirmed cardiac ion channel diseases (n = 3); (5) active chronic inflammation (n = 6); (6) continuous renal replacement therapy because of severe renal dysfunction with stage 5 of Chronic Kidney Disease (CKD-5) defined by KDOQI guideline^12^,^13^ (n = 4); (7) underlying in ischemic cardiomyopathy (n = 66); (8) or disorder in hematological and immunological system (n = 4). Design of this study and informed consent were approved by our local ethics committee (Ethics Committee of Zhongshan Hospital affiliated to Fudan University), and were carried out in accordance with the principles of the Declaration of Helsinki. Consent for publication of these data was obtained from each patient.

### CRT implantation

Patient met the indication for CRT implantation was firstly receiving selective coronary angiography to verify any lesion on coronary artery and optimal medical treatment for at least 3 months, then receiving CRT implantation if there was no improvement. The position of pacing lead was recorded in every patient. Left ventricular lead was targeted in coronary vein including posterio-lateral vein, posterior vein, lateral vein, major vein, and middle vein. Right ventricular lead was placed in apex or out flow tract of right chamber. These lead positions were chosen based on program control during operation. Besides, Patients were performed CRT optimization by UCG 3 months after implantation. AV delay optimization was performed by assessing the pattern of pulsed wave Doppler recorded through the mitral valve using the iterative method^14^. The optimal VV delay by the UCG was defined as the delay associated with the largest average the aortic velocity time integral (VTI). Aortic VTI measurements were obtained in accordance with the American Society of Echocardiography guidelines^15^.

### Electrocardiography

12-lead electrocardiograms (ECG) were performed in admission and within 48 hours after CRT implantation respectively. QRS duration, QT interval, corrected QT interval (QTc by Bazzet formula), T wave from peak to end interval (TpTe), ratio between TpTe and QTc (TpTe/QTc), and QRS morphology were measured through an electronic ECG analysis system by 2 independent cardiologists who didn’t know the study design and patients’ condition. When their opinions went against each other, another physician should be brought in to give an ultimate decision. For paroxysmal atrial fibrillation, the ECG without atrial fibrillation (AF) presentation were adopted. For persistent AF, parameter was calculated as mean value from measurement of 3 continuous complexes.

### Echocardiography

Echocardiograms (UCG) were performed within 1 month before and 1 year after CRT implantation in all patients using a Philips IE33 instrument (Philips, Netherlands) with a 2–3.5 MHz transducer (X3-1) to detect left ventricular ejection fraction (LVEF). Left atrial diameter (LAD), left ventricular end systolic diameter (LVESD), and left ventricular end diastolic diameter (LVEDD) were measure by M-mode. Left atrial volume (LAV), left ventricular end systolic volume (LVESV), and left ventricular end diastolic volume (LVEDV) were calculated by corrected Teichholz formula: V = 7.0/(2.4 + D) × D^3^ (V = LV volume, D = LV diameter) which was suitable for non-ischemic aetiology^16^. Pulmonary artery systolic pressure (PASP) was estimated from the tricuspid regurgitant jet velocity using the modified Bernoulli equation and adding the estimated right atrial pressure (RAP) on the basis of inferior vena cava (IVC) diameter and collapsibility according to the ASE guidelines for echocardiographic assessment of the right heart in adults: IVC diameter ≤2.1 cm that collapses >50% with a sniff suggests a normal RAP of 3 mm Hg (range, 0–5 mm Hg), whereas an IVC diameter >2.1 cm that collapses <50% with a sniff suggests a high RAP of 15 mm Hg (range, 10–20 mm Hg). In indeterminate cases in which the IVC diameter and collapse do not fit this paradigm, an intermediate value of 8 mm Hg (range, 5–10 mm Hg) was used^17^. Besides, E/A and E’/A were introduced as parameters reflecting cardiac diastolic function. E/A was the ratio of early and late peak values of mitral transvalvular blood flow speed through Doppler imaging. E’/A’ referred to the ratio of early and late peak values of mitral annulus tissue speed through Doppler imaging. S wave indicating the maximal velocity of mitral annulus motion revealed cardiac systolic function^18^,^19^. CRT response was defined as relative increase (≥15%) or absolute increase (≥10%) of LVEF after 1 year^20^,^21^. And if it didn’t meet the CRT response criteria, CRT non-response was defined. For paroxysmal AF, the UCG without atrial fibrillation presentation were adopted. For persistent AF, measurement was not different except for unavailability of E/A.

### Serology

Routine blood test items of HF patients in admission were collected from our medical record system, including serum creatinine (Scr), N-terminal Prohormone of Brain Natriuretic Peptide (NT-proBNP), troponine-T (cTnT), creatine kinase-MB (CK-MB) and C-reactive protein (CRP), etc. Furthermore, estimated glomerular filtration rate (eGFR) was calculated by MDRD formula and CKD-EPI formula based on the value of Scr and adjusted by patient’s age and sex^22^,^23^. MDRD formula was described as follows: eGFR for male = 175 × (Scr × 0.0113)^−1.154^ × age^−0.203^ and eGFR for female = 175 × (Scr × 0.0113)^−1.154^ × age^−0.203^ × 0.742, respectively. Well, CKD-EPI formula was more complicated because it was dependent on not only sex but also the range of Scr level, and the specific equations were depicted as below: for male with Scr ≤ 80: eGFR = 141 × (Scr × 0.0113/0.9)^−0.411^ × 0.993^age^, for male with Scr > 80: eGRF = 141 × (Scr × 0.0113/0.9)^−1.209^ × 0.993^age^, for female with Scr ≤ 62: eGFR = 144 × (Scr × 0.0113/0.7)^−0.329^ × 0.993^age^, and for female with Scr > 62: eGFR = 144 × (Scr × 0.0113/0.7)^−1.209^ × 0.993^age^. As these formulae mentioned above, the unit of eGFR was ml/min/1.73 m^2^, the unit of Scr was μmol/L, and the unit of age was year.

### Statistics

All statistical analyses were performed with SPSS software 19.0. Data were presented as the percentage, mean ± standard deviation (SD), or median values with their 25–75^th^ percentiles. Chi-square analysis was used to compare the frequency for categorical variables, Mann-Whitney test was used for ordinal categorical variables, and Student’s t tests were used to compare means for continuous variables. Multivariable logistic analysis was performed to identify the independent predictors for CRT non-response. All statistical analyses and graphs were performed using SPSS 19.0 software or Stata 12.0 software. All P-values were two-sided, and *P* < *0.05* was considered to indicate statistical significance.

### Ethics approval

This study was approved by the Ethics Committee of Zhongshan Hospital, Fudan University.

## Results

### Demographics and Baseline Characteristics

All 227 DHF patients enrolled with average 60.4 ± 12.3 years, including 163 men (71.8%). The prevalence of hypertension, diabetes, and atrial fibrillation (AF) were 40.1%, 18.1%, and 16.3% (30 paroxysmal AF and 7 persistent AF) respectively. Cardiac function (LVEF), renal function (Scr and eGFR), comorbidity (hypertension, diabetes, and AF), and medication prescriptions had no difference at baseline between CRT responders and CRT non-responders. Besides, markers of cardiac injury (cTnT, CK-MB, and NT-proBNP) showed equal levels in each group before CRT implantation. Baseline clinical characteristics of patients were shown in Table 1.

### Follow-up of the echocardiograph after 1 year

**Figure 1:**
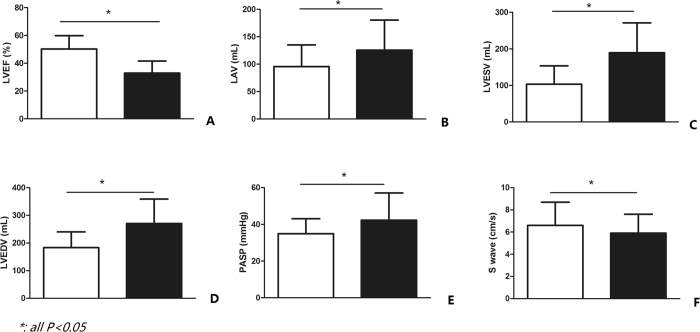
Indicated the difference of related UCG parameters between CRT responders and CRT non-responders. All bars in white color referred to CRT responders, while bars in black color represented for CRT non-responders. Besides, all of the p values here were less than 0.001 (CRT responders vs CRT non-responders).

UCG data after 1 year of CRT implantation were collected properly, and patients according with inclusion criteria were then separated into two groups, CRT responders and CRT non-responders respectively, based on the above mentioned definition of CRT response. LVEF was significantly increased in CRT responders (50.2 ± 9.7% vs 32.9 ± 8.7%, *p* < *0.001*, Fig. 1A). Compared to CRT responders whose mean value of LAD was 44.7 ± 7 mm, the mean value of CRT non-responders was 50.2 ± 8.9 mm (*p* < *0.001*). Additionally, the mean value of LVESD was higher in CRT non-responders than CRT responders (44.7 ± 9.8 mm vs 61.3 ± 19.9 mm, *p* < *0.001*). Moreover, in comparison with CRT responders, the value of LVEDD was higher in CRT non-responders (59.2 ± 8.2 mm vs 70.9 ± 9.9 mm, *p* < *0.001*). The value of LAV, LVESV, and LVEDV were higher in CRT non-responders (95.8 ± 39.3 vs 125.5 ± 55 mL, 103.3 ± 50.3 vs 189.6 ± 81.7 mL, and 183.2 ± 57.1 vs 270.4 ± 88.7 mL respectively, *p* < *0.001*, Fig. 1B,C,D). Besides, the mean value of PASP in CRT responders was significantly lower than CRT non-responders’ (34.9 ± 8.1 mmHg vs 42.3 ± 14.8 mmHg, *p* < *0.001*, Fig. 1E). In addition for S wave, the summit velocity of mitral annulus’ motion in CRT non-responders was distinctly slower than the one in CRT responders (6.6 ± 2.1 cm/s vs 5.9 ± 1.7 cm/s, *p* = *0.012*, Fig. 1F). Scr level was lower in CRT responders (75.2 ± 23.4 μmol/L vs 92.5 ± 30.1 μmol/L, Fig. 1).

### Analysis of CRT non-response predictors

#### Univariate analysis

**Figure 2:**
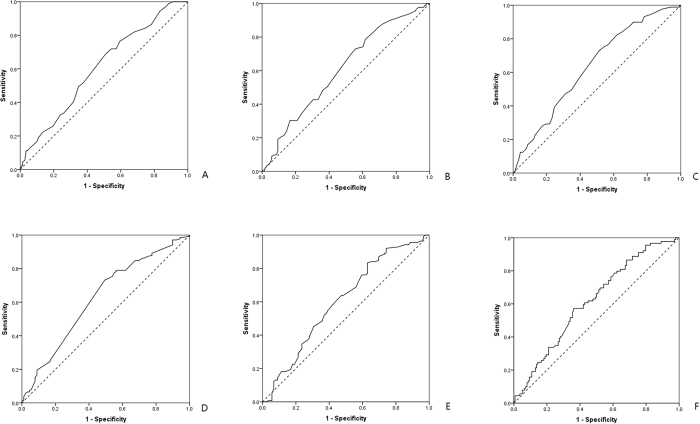
Showed ROC curve to predict the optimal cut off of CRT non-response predictors, and all p values were less than 0.01. The optimal cut-off point was in the upper-left area and was calculated based on the maximal Youden index (sensitivity + specificity − 1). (**A**) Was ROC curve of LAV; (**B**) was ROC curve of LVESV; (**C**) was ROC curve of LVEDV; (**D**) was ROC curve of S wave; (**E**) was ROC curve of QRS duration; (**F**) was ROC curve of TpTe/QTc.

**Figure 3:**
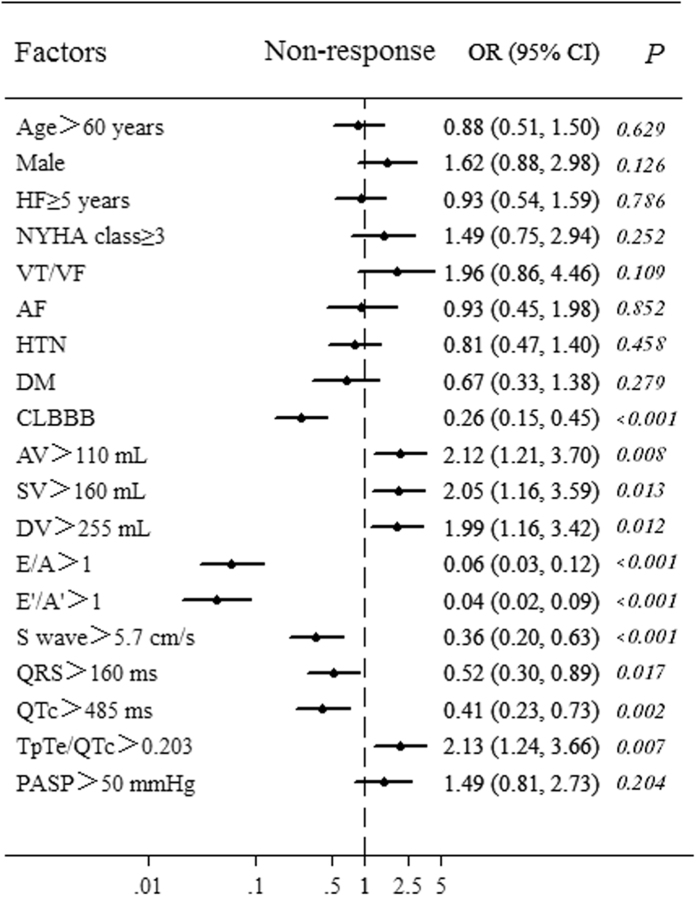
Showed univariate logistic regression analysis of electro-echocardiographic indices and other clinical factors for predicting CRT non-response. Odds ratio (OR) and its 95% credential interval (CI) of each factor was presented as point and line respectively. Risk of CTR non-response increased when OR > 1, while it decreased when OR < 1. HF = heart failure; NYHA = New York Heart Association; AF = atrial fibrillation; HTN = hypertension; DM = diabetes mellitus; AV = left atrial volume; SV = left ventricular end systolic volume; DV = left ventricular end diastolic volume; CLBBB = complete left bundle branch block.

Univariate analysis was performed to preliminarily filter risk factors of CRT non-response. The optimal cut-off points of risk predictors were selected by receiver operating characteristic (ROC) curve based on the maximal Youden index (sensitivity + specificity − 1) or median. ROC analysis was applied to electro-echocardiographic indices (Fig. 2). Area under the curve (AUC) and the optimal cut-off point’s sensitivity and specificity were shown in Table 2. LAV > 110 mL [*Odds Ratio (OR*) = *2.116, p* *=* *0.008*], LVESV > 160 mL (*OR* = *2.045, p* = *0.013*), and LVEDV > 255 mL (*OR* = *1.994, p* = *0.012*) showed great potential to predict CRT non-response. Besides, S wave > 5.7 cm/s (*OR* = *0.358, p* < *0.001*), E/A > 1 (*OR* = *0.059, p* < *0.001*), E’/A’ > 1 (*OR* = *0.043, p* < *0.001*), QRS > 160 ms (*OR* = *0.517, p* = *0.017*), QTc > 485 ms (*OR* = *0.413, p* = *0.002*), and TpTe/QTc > 0.203 (*OR* = *0.216, p* = *0.007*), as well as CLBBB (*OR* = *0.258, p* < *0.001*) a widely known CRT response predictor, indicated low-probability of CRT non-response. However, HF history, NYHA class, comorbidity of AF, hypertension, and diabetes, and PASP failed to predict CRT non-response (Fig. 3).

#### Multivariate analysis

Multivariate logistic analysis was performed to demonstrate the independent effect of these predictors (confirmed statistic difference in univariate analysis) on the occurrence of CRT non-response. Moreover, since previous studies reported age, sex, and NYHA class^5^ were related to CRT response, these factors were included in multivariate analysis to correct the latent bias. In this analysis, CRT non-response was employed as a dependent variable, while CLBBB, LAV > 110 mL, LVESV > 160 mL, LVEDV > 255 mL, S wave > 5.7 cm/s, E/A > 1, E’/A’ > 1, QRS > 160 ms, QTc > 485 ms, TpTe/QTc > 0.203, age > 60 years, male, and NYHA class > III were set as independent variables. LVEDV > 255 mL (*OR* = *2.236; 95*% *CI, 1.016*–*4.923*) rather than LVESV > 160 mL (*OR* = *1.18; 95*% *CI, 0.544*–*2.56*) and TpTe/QTc > 0.203 (*OR* = *5.206; 95*% *CI, 1.89*–*14.34*) significantly predicted CRT non-response. Oppositely, S wave > 5.7 cm/s (*OR* = *0.242; 95*% *CI, 0.089*–*0.657*), E/A > 1 (*OR* = *0.211; 95*% *CI, 0.079*–*0.566*), E’/A’ > 1 (*OR* = *0.054; 95*% *CI, 0.017*–*0.172*), CLBBB (*OR* = *0.141; 95*% *CI, 0.048*–*0.409*), and QRS duration >160 ms (*OR* = *0.52; 95*% *CI, 0.305*–*0.922*) surprisingly predicted low-probability of CRT non-response (Table 3).

## Discussion

Existed researches showed ischemic aetiology, female, and non-CLBBB, etc. are related to CRT non-response^5^,^24^, while it still lacks of credible and efficient indices to predict CRT non-response since there exist amount of non-responders to CRT (almost 30%) with unclear reasons^2^. The present study indicated higher values of TpTe/QTc and LVEDV but not LAV or LVESV were promising to independently predict CRT non-response. While, higher values of S wave and QRS duration, E/A > 1, E’/A’ > 1 at baseline predicted lower possibility of CRT non-response except for the traditional predictor CLBBB. Oppositely, QTc failed to independently predict CRT non-response though it showed significant difference in single-variate analysis. We thought QTc interval should be dependent on QRS duration. Results in this study are partly consistent with prior studies^25^,^26^, for example LVEDD, LVEDV and non-CLBBB, however, TpTe/QTc, E/A, E’/A’, and S wave as risk factors predicting CRT non-response were seldom reported before. PASP can reflect the function of right ventricle^25^,^27^ and the pumping function is normally maintained by synchronous movement between left and right ventricular walls. Besides, increased PASP brought problems to pulmonary leading to hypoxemia which aggravating oxygen deficit of cardiomyocyte. Thus, abnormal PASP related to devastating cardiac function at baseline predicted CRT non-response However, in present study, PASP estimated by empirical equation failed to be filtered through single-variate analysis. That may be related to the limited sample size and the way of ultrasonic Doppler to measure PASP, because Doppler could underestimate PASP if there was no tricuspid regurgitation being detected. In addition, parameters reflecting left ventricular diastolic function such as LVEDV, E/A, and E’/A’ showed close relationship with CRT non-response^18^,^19^. As is known to us, heart failure with impaired ejection fraction is indicated for CRT. However, it is thought-provoking that LVEDV rather than LVESV predicted CRT non-response in this study. To some extent, LVEDV reflected diastolic function which was crucial for cardiac filling volume, while LVESV determined pumping volume. Studies showed that instead of systolic function, clinical prognosis post-CRT implantation was predicted by diastolic function^28^,^29^,^30^. Badly impaired diastolic function was hardly reversed by CRT, so that LVEDV played important roles to predict CRT non-response. On the one hand, LVESV were important to evaluate the effect of CRT; while on the other hand, LVEDD and LVEDV were valuable for predicting CRT non-response^30^. What’s more, E/A indicating the condition of blood flow over mitral valve was valuable for CRT non-response prediction, but E’/A’ referring to the motion of mitral annulus was insignificant. As mentioned above, CRT was especially effective in dyssynchronous HF, and it was reported that E/A can predict CRT response within 6 months^31^, however, little was known about the effect of E’/A’ and S wave. Present study showed the value of S wave and E’/A’ to predict CRT non-response.

**Figure 4:**
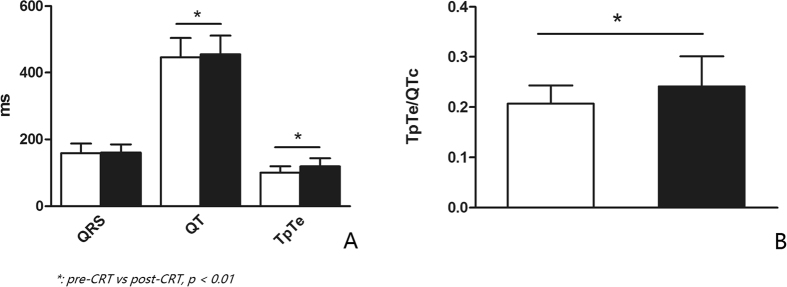
Was the comparison of electrocardiographic indice about dispersion of repolarization between pre-CRT placement and post-CRT placement. (**A**) and (**B**) showed QT interval, TpTe and TpTe/QTc significantly increased within 3 days after CRT implantation indicating CRT prompting TDR increasing in early stage (white bars represented for indices of pre-CRT, and black bars referred to indices of post-CRT).

It was reported that both QTc and TpTe/QTc reflect the degree of cardiac transmural dispersion of repolarization (TDR)^32^,^33^. Shorter QTc predicted CRT non-response increment. On the contrary, smaller TpTe/QTc value predicted CRT-nonresponse reduction. Unfortunately, TpTe was not statistically significant in predicting CRT non-response, so the effect of TpTe/QTc in this study may be largely determined by QTc. Interestingly, QTc interval is related to the degree of TDR, and the increase of TDR is underlying arrhythmogenesis^34^. Thus, shorter QTc interval indicating less TDR should do good. However, present study showed shorter QTc as a harmful factor, having high risk in CRT non-response. It seemed QTc as a double-edged sword increased arrhythmia when lengthening, but decreased CRT non-response when shortening. Moreover, CRT changes the direction of cardiac repolarization, and it could contribute to the increase of TDR. Under physiological condition, hearts depolarize from endocardium to epicardium, and repolarize in an opposite direction^35^. However, CRT totally changes left ventriclular direction of depolarization and repolarization since its epicardial pacing, leading to TDR increasing^36^. It was reported that occurrence of tachycardia was higher in early stage of CRT implantation^37^. In this study, compared to baseline, TpTe and QT which reflect the degree of TDR were significantly increased within 72 h after CRT implantation (100 ± 20 ms vs. 119 ± 24 ms, and 446 ± 58 ms vs. 455 ± 56 ms respectively, p < 0.01, indicating that CRT increases the dispersion of repolarization and has potential to cause arrhythmia in the early stage (Fig. 4). It indicates that cardiac dispersion of repolarization may have dual effect on patients with CRT.

**Figure 5:**
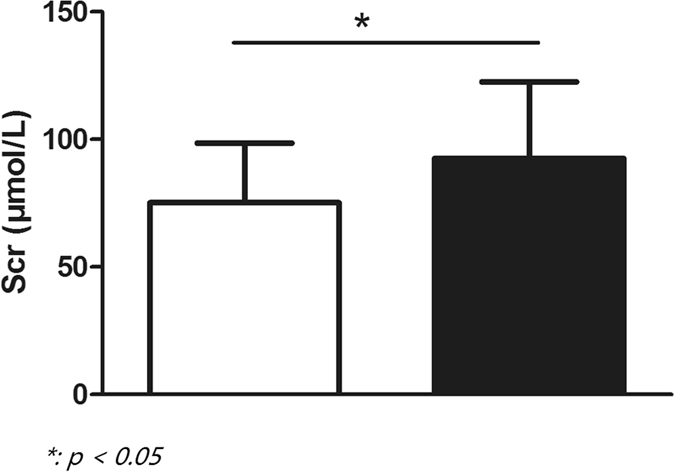
Showed difference of Scr level between CRT responders and CRT non-responders after 1 year. Bar in white color referred to CRT responders, while bar in black color represented for CRT non-responders.

Since severe renal dysfunction could be a confounding factor, and end stage renal disease is always complicated with multiple-organ disorder including heart problems. Thus, we excluded patients in CKD-5 with continuous renal replacement therapy. Scr can somewhat reflect cardiac function since cardiac dysfunction resulting in low pumping volume could directly affect the perfusion of kidney, thus Scr could predict prognosis of HF^38^. What’s more, chronic kidney disease could influence CRT non-response as concomitant disease^39^. However, oppositely, lower level of Scr, reflecting muscle mass decreasing, was related to poor prognosis in HF^40^. It seemed that on the one hand, increased Scr indicating renal function worsening was in relationship with poor prognosis after CRT implantation; while on the other hand, decreased Scr indicating over self-consumption prognosed bad outcome. Considering renal function might be related to CRT non-response, we analyzed both the value of Scr and eGFR. However, at baseline, there was no difference between CRT responders and CRT non-responders. While, after one year of CRT implantation, Scr level of CRT responders was significantly lower than CRT non-responders’ (Fig. 5). It hinted that Scr or eGFR might be more suitable for evaluation the effect of CRT.

Limitations of our study included a retrospective design, single centre participation and short study duration. Thus, a prospective, large scale, single blind to patients, randomized, controlled, and multi-center collaborative clinical trial is still in high need in future. Besides, other valuable parameters such as left ventricular volume, E’, and E/E’ were not included because they were not available with a retrospective design.

## Conclusion

The present study revealed that both electrocardiographic and echocardiographic indice, especially TpTe/QTc > 0.203, and LVEDV > 255 mL are valuable to predict CRT non-response. CLBBB, QRS duration >160 ms, S wave >5.7 cm, E/A > 1, and E’/A’ > 1 showed lower probability of CRT non-response. The findings of this study provide some useful parameters on predicting CRT non-response. However the sample size in this study is not large enough, though 227 objects included for single-center in this area is less common. Consequently, large scale, multi-center, and prospective research is highly wanted in future.

## Additional Information

**How to cite this article**: Yu, Z. *et al*. Electro-echocardiographic Indices to Predict Cardiac Resynchronization Therapy Non-response on Non-ischemic Cardiomyopathy. *Sci. Rep.* **7**, 44009; doi: 10.1038/srep44009 (2017).

**Publisher's note:** Springer Nature remains neutral with regard to jurisdictional claims in published maps and institutional affiliations.

## Figures and Tables

**Table 1:** Comparison of clinical characteristics between CRT response and CRT non-response HF patients in baseline.

Factors	Total	Response (n = 138)	non-Response (n = 89)	*p* value
(**1**) **Demography**				
Age (years)	60.4 ± 12.3	61.2 ± 12.6	59.3 ± 11.5	*0.266*
Male n, (%)	163 (71.8)	94 (68.1)	69 (77.5)	*0.124*
NYHA	2.9 ± 0.5	2.9 ± 0.6	2.9 ± 0.5	*0.334*
II n, (%)	47 (20.7)	32 (23.2)	15 (16.9)	
III n, (%)	158 (69.6)	93 (67.4)	65 (73)	
IV n, (%)	22 (9.7)	13 (9.4)	9 (10.1)	
DM n, (%)	41 (18.1)	28 (20.3)	13 (14.6)	*0.277*
HTN n, (%)	91 (40.1)	58 (42)	33 (37.1)	*0.457*
VT/VF history n, (%)	26 (11.5)	12 (8.7)	14 (15.7)	*0.104*
HF history (years)	4.8 ± 5.2	4.8 ± 5.3	4.6 ± 5	*0.827*
AF n, (%)	37 (16.3)	23 (16.7)	14 (15.7)	*0.852*
Hemoglobin (g/L)	132.1 ± 19.5	131.9 ± 17.6	132.4 ± 22.1	*0.863*
Albumin (g/L)	38.9 ± 3.6	38.8 ± 3.6	39 ± 3.7	*0.386*
(**2**) **Echocardiography**				
LVEF (%)	30 ± 7	30.3 ± 6.2	32.5 ± 8.7	*0.084*
PASP (mmHg)	44.4 ± 13.9	43.5 ± 13	45.8 ± 15.1	*0.23*
LAD (mm)	48.9 ± 7.1	47.9 ± 7	50.6 ± 7	*0.005*
LVESD (mm)	58.6 ± 9.4	57.3 ± 9.6	60.6 ± 8.7	*0.01*
LVEDD (mm)	70.3 ± 8.4	68.9 ± 8.4	72.6 ± 7.9	*0.001*
LAV (mL)	116 ± 42	110.3 ± 39.9	124.8 ± 44	*0.011*
LVESV (mL)	176.5 ± 64	168.4 ± 64.5	189.2 ± 61.5	*0.015*
LVEDV (mL)	263.1 ± 70.9	251.3 ± 69.6	281.3 ± 69.2	*0.002*
S wave (cm/s)	5.6 ± 1.4	5.8 ± 1.3	5.3 ± 1.3	*0.003*
E/A > 1 n, (%)	122 (53.7)	107 (77.5)	15 (16.9)	*＜0.001*
E’/A’ > 1 n, (%)	120 (52.9)	108 (78.3)	12 (13.5)	*＜0.001*
(**3**) **Electrocardiography**				
CLBBB n, (%)	136 (59.9)	100 (72.5)	36 (40.4)	*＜0.001*
QRS duration (ms)	160.8 ± 35.8	163.7 ± 25.9	156.3 ± 47.1	*0.178*
QTc (ms)	481.9 ± 52.4	491 ± 41.1	472.8 ± 40	*0.001*
TpTe (ms)	97.7 ± 19.5	96.4 ± 17.5	99.7 ± 22.1	*0.211*
TpTe/QTc	0.202 ± 0.037	0.196 ± 0.034	0.211 ± 0.039	*0.005*
(**4**) **Serology**				
Serum potassium (mmol/L)	4.1 ± 0.4	4.1 ± 0.4	4.1 ± 0.5	*0.943*
CRP (mg/L)	12.3 ± 19	13.4 ± 23.3	10.7 ± 9	*0.31*
Serum creatinine (μmol/L)	94.4 ± 33.4	92.5 ± 31.5	97.3 ± 36.2	*0.291*
eGFR (ml/min/1.73 m^2^, calculated by 2 different equations)				
by MDRD formula	72.4 ± 23.1	73.4 ± 23.6	71 ± 22.4	*0.439*
by CKD-EPI formula	74.1 ± 21.2	74.6 ± 21.3	73.4 ± 21.2	*0.663*
NT-proBNP (pg/mL)	4505.5 ± 4738.9	4196.5 ± 4343.7	4984.6 ± 5283.7	*0.222*
Troponin T (ng/mL)	0.059 ± 0.167	0.056 ± 0.174	0.064 ± 0.157	*0.723*
CK-MB (U/L)	12.4 ± 4.7	12.7 ± 5.4	12.1 ± 3.2	*0.299*
(**5**) **Medication**				
ACEI/ARB n, (%)	189 (83.3)	115 (83.3)	74 (83.1)	*0.971*
β- blocker (%)	183 (80.6)	108 (78.3)	75 (84.3)	*0.264*
Spironolactone n, (%)	188 (82.8)	116 (84.1)	72 (80.9)	*0.538*
Digoxin n, (%)	104 (45.8)	65 (47.1)	39 (43.8)	*0.628*
Amiodorane n, (%)	39 (17.2)	21 (15.2)	18 (20.2)	*0.329*
Loop-diuretics n, (%)	179 (78.9)	108 (78.3)	71 (79.8)	*0.785*

Indicated clinical characteristics between CRT response and CRT non-response HF patients before CRT implantation. From this table, age, sex, concomitant disease, HF history, baseline EF, electrolyte and other bio-marker, and medication were not different between two groups. However, indice of UCG and ECG were significantly different.

**Table 2:** Optimal cut-off points and related diagnostic value by ROC analysis.

Factors	Cut-off points	Sensitivity	Specificity	AUC	*P* value
LAV	110 mL	0.685	0.493	0.606	*0.007*
LVESV	160 mL	0.73	0.442	0.606	*0.007*
LVEDV	255 mL	0.73	0.486	0.632	*0.001*
S wave	5.7 cm/s	0.732	0.506	0.626	*0.001*
QRS_duration_	160 ms	0.833	0.371	0.603	*0.009*
QTc_interval_	485 ms	0.522	0.697	0.631	*0.001*
TpTe/QTc	0.203	0.573	0.638	0.616	*0.003*

Showed optimal cut-off points and related diagnostic value by ROC analysis with related sensitivity, specificity, AUC, and p value (LAV = left atrial volume; LVESV = left ventricular end systolic volume; LVEDV = left ventricular end diastolic volume; AUC = area under the curve). Larger AUC indicated better diagnostic value.

**Table 3:** Odds ratios of independent predictors for CRT non-response in HF patients (multivariate logistic analysis).

Predictors	Odds Ratio	95% confidence intervals	*p* value
Age >60 years	0.917	0.527–1.596	*0.759*
Male	1.643	0.885–3.05	*0.116*
HF ≥ 5 years	0.872	0.506–1.506	*0.624*
NYHA class ≥ 3	1.563	0.782–3.126	*0.206*
AF	0.892	0.427–1.864	*0.761*
HTN	0.794	0.451–1.506	*0.425*
DM	0.543	0.167–1.769	*0.311*
LAV > 110 mL	1.001	0.987–1.015	*0.889*
LVESV > 160 mL	1.18	0.544–2.56	*0.676*
LVEDV > 255 mL	2.236	1.016–4.923	*0.046*
E/A > 1	0.211	0.079–0.566	*0.002*
E’/A’ > 1	0.054	0.017–0.172	*＜0.001*
S > 5.7 cm/s	0.242	0.089–0.657	*0.005*
CLBBB	0.141	0.048–0.409	*＜0.001*
QRS > 160 ms	0.53	0.305–0.922	*0.025*
QTc > 485 ms	0.539	0.189–1.535	*0.247*
TpTe/QTc > 0.203	5.206	1.89–14.34	*0.001*

Presented multivariate logistic regression analysis of risk factors with odds ratio and its 95% confidence interval. LVEDV but not LVESV, TpTe/QTc, CLBBB, QRS, E/A, E’/A’ and S wave were valuable to predict CRT non-response.
